# Supplementary material for: Grit (effortful persistence) can be measured with a short scale, shows little variation across socio-demographic subgroups, and is associated with career success and career engagement
Source: PLoS One. 2019 Nov 27;14(11):e0224814. doi: 10.1371/journal.pone.0224814 (PMC6881019; doi:10.1371/journal.pone.0224814)
Supplement: S1 Table — (DOCX) [file pone.0224814.s001.docx]

**S1 Table. Descriptive Statistics for Study 1.**

| Variable | *N* | Min. | Max. | *M* | *SD* |
| --- | --- | --- | --- | --- | --- |
| Age in years | 6,231 | 17 | 90 | 42.64 | 14.47 |
| Gender (1 = *female*, 0 = *male*) | 6,231 | 0 | 1 | 0.51 | 0.50 |
| Educational attainment |  |  |  |  |  |
| lower (CASMIN 1–3) | 6,024 | 0 | 1 | 0.27 | 0.44 |
| intermediate (CASMIN 4–7) | 6,024 | 0 | 1 | 0.51 | 0.50 |
| higher (CASMIN 8–9) | 6,024 | 0 | 1 | 0.22 | 0.42 |
| Employed (1 = *yes*, 0 = *no*) | 6,165 | 0 | 1 | 0.63 | 0.48 |
| Grit scale |  |  |  |  |  |
| Item 1: “I am a hard worker“ | 6,213 | 1 | 5 | 3.70 | 0.82 |
| Item 2: “I am diligent” | 6,221 | 1 | 5 | 3.68 | 0.78 |
| Item 3: “I can cope with setbacks” | 6,212 | 1 | 5 | 3.24 | 0.80 |
| Item 4: “I finish whatever I begin” | 6,229 | 1 | 5 | 4.11 | 0.73 |
| Item 5: “I have difficulty maintaining focus…” | 6,189 | 1 | 5 | 2.29 | 1.00 |
