# Supplementary material for: Grit (effortful persistence) can be measured with a short scale, shows little variation across socio-demographic subgroups, and is associated with career success and career engagement
Source: PLoS One. 2019 Nov 27;14(11):e0224814. doi: 10.1371/journal.pone.0224814 (PMC6881019; doi:10.1371/journal.pone.0224814)
Supplement: S2 Table — (DOCX) [file pone.0224814.s002.docx]

**S2 Table. Zero-Order Correlations for All Study Variables (Study 1).**

|  |  |  |  |  |  |  |  |  |  |  |  |
| --- | --- | --- | --- | --- | --- | --- | --- | --- | --- | --- | --- |
| # | Variable | 1 | 2 | 3 | 4 | 5 | 6 | 7 | 8 | 9 | 10 |
| 1 | Age in years |  |  |  |  |  |  |  |  |  |  |
| 2 | Gender (1 = *female*, 0 = *male*) | –.02 |  |  |  |  |  |  |  |  |  |
|  | Educational attainment |  |  |  |  |  |  |  |  |  |  |
| 3 | lower (CASMIN 1–3) | **.19** | **–.08** |  |  |  |  |  |  |  |  |
| 4 | intermediate (CASMIN 4–7) | **–.23** | **.10** | **–.62** |  |  |  |  |  |  |  |
| 5 | higher (CASMIN 8–9) | **.07** | **–.04** | **–.32** | **–.54** |  |  |  |  |  |  |
| 6 | Employed (1 = *yes*, 0 = *no*) | –.01 | **–.17** | **–.13** | .02 | **.12** |  |  |  |  |  |
|  | Grit scale |  |  |  |  |  |  |  |  |  |  |
| 7 | Item 1: “I am a hard worker“ | **–.05** | **–.06** | **–.05** | .00 | **.05** | **.27** |  |  |  |  |
| 8 | Item 2: “I am diligent” | **.04** | –.03 | –.02 | –.03 | **.06** | **.11** | **.43** |  |  |  |
| 9 | Item 3: “I can cope with setbacks” | –.01 | **–.17** | **–.06** | .00 | **.06** | **.09** | **.19** | **.26** |  |  |
| 10 | Item 4: “I finish whatever I begin” | .03 | –.00 | –.02 | –.01 | **.03** | **.09** | **.31** | **.39** | **.19** |  |
| 11 | Item 5: “I have difficulty maintaining focus…” | **–.05** | .03 | **.10** | .01 | **–.12** | **–.13** | **–.17** | **–.21** | **–.18** | **–.24** |

*Note*. Coefficients are Pearson correlation coefficients (*r*) with pairwise deletion. Values printed in boldface are statistically significant at *p* < .01.
