# Supplementary material for: Grit (effortful persistence) can be measured with a short scale, shows little variation across socio-demographic subgroups, and is associated with career success and career engagement
Source: PLoS One. 2019 Nov 27;14(11):e0224814. doi: 10.1371/journal.pone.0224814 (PMC6881019; doi:10.1371/journal.pone.0224814)
Supplement: S3 Table — (DOCX) [file pone.0224814.s003.docx]

**S3 Table. Descriptive Statistics for Study 2.**

| Variable | *N* | Min. | Max. | *M* | *SD* |
| --- | --- | --- | --- | --- | --- |
| Age in years | 2,246 | 18 | 68 | 42.09 | 12.19 |
| Gender (1 = *female*, 0 = *male*) | 2,246 | 0 | 1 | 0.44 | 0.50 |
| Educational attainment |  |  |  |  |  |
| lower (CASMIN 1–3) | 2,244 | 0 | 1 | 0.22 | 0.41 |
| intermediate (CASMIN 4–7) | 2,244 | 0 | 1 | 0.51 | 0.50 |
| higher (CASMIN 8–9) | 2,244 | 0 | 1 | 0.28 | 0.45 |
| Employment (1 = *full-time*, 0 = *part-time*) | 2,246 | 0 | 1 | 0.75 | 0.43 |
| Cognitive ability | 2,246 |  |  |  |  |
| Literacy | 2,246 | 74.2 | 457.0 | 283.8 | 40.6 |
| Numeracy | 2,246 | 87.6 | 486.3 | 288.5 | 45.5 |
| Problem solving | 2,032 | 73.1 | 411.7 | 283.8 | 40.0 |
| Grit |  |  |  |  |  |
| Item 1: “I am a hard worker“ | 2,245 | 1 | 5 | 3.85 | 0.75 |
| Item 2: “I am diligent” | 2,244 | 1 | 5 | 3.73 | 0.75 |
| Item 3: “I can cope with setbacks” | 2,240 | 1 | 5 | 3.30 | 0.77 |
| Item 4: “I finish whatever I begin” | 2,245 | 2 | 5 | 4.16 | 0.69 |
| Item 5: “I have difficulty maintaining focus…” | 2,235 | 1 | 5 | 2.19 | 0.94 |
| Conscientiousness |  |  |  |  |  |
| Item 1: “…works thoroughly” | 2,246 | 1 | 7 | 6.24 | 0.90 |
| Item 2: “…is rather lazy” | 2,245 | 1 | 7 | 2.58 | 1.65 |
| Item 3: “…carries out duties efficiently” | 2,244 | 1 | 7 | 5.90 | 0.91 |
| Career Success and engagement |  |  |  |  |  |
| Income (EUR / month) | 2,216 | 30 | 96,000 | 2,958 | 2766,45 |
| Job prestige (SIOPS) | 2,072 | 13.00 | 78.16 | 45.79 | 13.34 |
| Job satisfaction | 2,209 | 0 | 10 | 7.44 | 2.03 |
| N. of hours overtime / week (past month) | 1,984 | 0 | 40 | 4.00 | 5.21 |
| N. of CPD courses (past year) | 2,240 | 0 | 20 | 1.23 | 2.25 |
| Learning orientation | 2,244 | 1 | 7 | 6.53 | 0.86 |

*Note*. Unweighted sample values. In subsequent analyses, post-stratification weights were applied.
