# Supplementary material for: Grit (effortful persistence) can be measured with a short scale, shows little variation across socio-demographic subgroups, and is associated with career success and career engagement
Source: PLoS One. 2019 Nov 27;14(11):e0224814. doi: 10.1371/journal.pone.0224814 (PMC6881019; doi:10.1371/journal.pone.0224814)
Supplement: S4 Table — (DOCX) [file pone.0224814.s004.docx]

**S4 Table. Zero-Order Correlations for All Study Variables (Study 2).**

|  | *Variable* | 1 | 2 | 3 | 4 | 5 | 6 | 7 | 8 | 9 | 10 | 11 |
| --- | --- | --- | --- | --- | --- | --- | --- | --- | --- | --- | --- | --- |
| 1 | Age in years |  |  |  |  |  |  |  |  |  |  |  |
| 2 | Gender (1 = *female*, 0 = *male*) | .03 |  |  |  |  |  |  |  |  |  |  |
| 3 | Education: lower (CASMIN 1–3) | **.09** | **–.14** |  |  |  |  |  |  |  |  |  |
| 4 | Education: intermediate (CASMIN 4–7) | **–.15** | **.11** | **–.53** |  |  |  |  |  |  |  |  |
| 5 | Education: higher (CASMIN 8–9) | **.09** | .01 | **–.33** | **–.63** |  |  |  |  |  |  |  |
| 6 | Employment (1 = *full-time*) | **.10** | **–.35** | **.06** | **–.11** | **.07** |  |  |  |  |  |  |
| 7 | Cognitive ability: Literacy | **–.09** | .02 | **–.44** | .01 | **.39** | .02 |  |  |  |  |  |
| 8 | Cognitive ability: Numeracy | –.02 | **–.10** | **–.39** | –.05 | **.41** | **.11** | **.87** |  |  |  |  |
| 9 | Cognitive ability: Problem solving | **–.21** | –.04 | **–.41** | –.01 | **.37** | .04 | **.82** | **.77** |  |  |  |
| 10 | Grit 1: “I am a hard worker“ | –.03 | –.01 | .03 | –.03 | .01 | **.10** | **–.08** | –.05 | **–.06** |  |  |
| 11 | Grit 2: “I am diligent” | .03 | –.01 | –.01 | –.03 | .04 | .04 | –.04 | –.01 | **–.08** | **.42** |  |
| 12 | Grit 3: “I can cope with setbacks” | .03 | **–.13** | –.04 | –.01 | .05 | **.07** | .04 | .07 | –.01 | **.17** | **.26** |
| 13 | Grit 4: “I finish whatever I begin” | .02 | .00 | .04 | –.05 | .02 | .01 | **–.07** | –.05 | **–.10** | **.29** | **.37** |
| 14 | Grit 5: “I have difficulty maintaining focus” | **–.08** | .00 | **.07** | .03 | **–.11** | –.01 | **–.09** | **–.12** | –.05 | **–.11** | **–.19** |
| 15 | Consc. 1: “works thoroughly” | **.07** | **.11** | **.07** | .03 | **–.10** | –.01 | **–.11** | **–.12** | **–.13** | **.28** | **.30** |
| 16 | Consc. 2: “is rather lazy” | **–.14** | **–.10** | **–.07** | .02 | .04 | –.00 | **.14** | **.12** | **.17** | **–.28** | **–.28** |
| 17 | Consc.: 3 “carries out duties efficiently” | .04 | .05 | .05 | .01 | **–.06** | .03 | **–.09** | **–.08** | **–.12** | **.29** | **.34** |
| 18 | Income (EUR / month) | **.18** | **–.18** | **–.08** | **–.16** | **.25** | **.30** | **.21** | **.25** | **.15** | **.07** | **.08** |
| 19 | Job prestige (SIOPS) | .03 | **.11** | **–.32** | **–.18** | **.50** | .02 | **.46** | **.43** | **.38** | –.02 | .05 |
| 20 | Job satisfaction | –.03 | .03 | .00 | .01 | –.01 | –.05 | .00 | –.01 | –.03 | **.06** | **.12** |
| 21 | N. of hours overtime | –.01 | **–.10** | –.02 | **–.10** | **.14** | **.10** | .04 | **.08** | **.06** | **.24** | **.12** |
| 22 | N. of CPD courses | **.08** | **.09** | **–.11** | –.03 | **.14** | –.01 | **.12** | **.11** | **.07** | **.06** | .03 |
| 23 | Learning orientation | **.06** | .03 | **–.10** | –.01 | **.10** | .02 | **.12** | **.10** | **.06** | **.10** | **.10** |

**S4 Table (continued).**

|  | *Variable* | 12 | 13 | 14 | 15 | 16 | 17 | 18 | 19 | 20 | 21 | 22 |
| --- | --- | --- | --- | --- | --- | --- | --- | --- | --- | --- | --- | --- |
| 13 | Grit 4: “I finish whatever I begin” | **.18** |  |  |  |  |  |  |  |  |  |  |
| 14 | Grit 5: “I have difficulty maintaining focus…” | **–.17** | **–.24** |  |  |  |  |  |  |  |  |  |
| 15 | Consc. 1: “…works thoroughly” | **.08** | **.32** | **–.15** |  |  |  |  |  |  |  |  |
| 16 | Consc. 2: “…is rather lazy” | **–.03** | **–.28** | **.18** | **–.32** |  |  |  |  |  |  |  |
| 17 | Consc.: 3 “…carries out duties efficiently” | **.13** | **.38** | **–.22** | **.48** | **–.26** |  |  |  |  |  |  |
| 18 | Income (EUR / month) | **.10** | .04 | **–.09** | –.03 | –.01 | .02 |  |  |  |  |  |
| 19 | Job prestige (SIOPS) | .04 | **.06** | **–.13** | –.05 | .04 | .01 | **.25** |  |  |  |  |
| 20 | Job satisfaction | **.12** | **.11** | **–.13** | **.12** | **–.09** | **.12** | .04 | **.07** |  |  |  |
| 21 | N. of hours overtime / week (past month) | .04 | **.10** | **–.06** | .00 | –.06 | **.06** | **.24** | **.13** | –.00 |  |  |
| 22 | N. of CPD courses (past year) | –.01 | .05 | **–.08** | .03 | **–.07** | **.03** | **.12** | **.21** | **.08** | **.07** |  |
| 23 | Learning orientation | **.06** | **.14** | **–.12** | **.11** | **–.10** | **.15** | **.06** | **.15** | **.13** | .05 | **.14** |

*Note*. Coefficients are Pearson correlation coefficients (*r*) with pairwise deletion. Values printed in boldface are statistically significant at *p* < .01. Consc. = conscientiousness.
