# Supplementary material for: Grit (effortful persistence) can be measured with a short scale, shows little variation across socio-demographic subgroups, and is associated with career success and career engagement
Source: PLoS One. 2019 Nov 27;14(11):e0224814. doi: 10.1371/journal.pone.0224814 (PMC6881019; doi:10.1371/journal.pone.0224814)
Supplement: S1 Fig — (DOCX) [file pone.0224814.s005.docx]

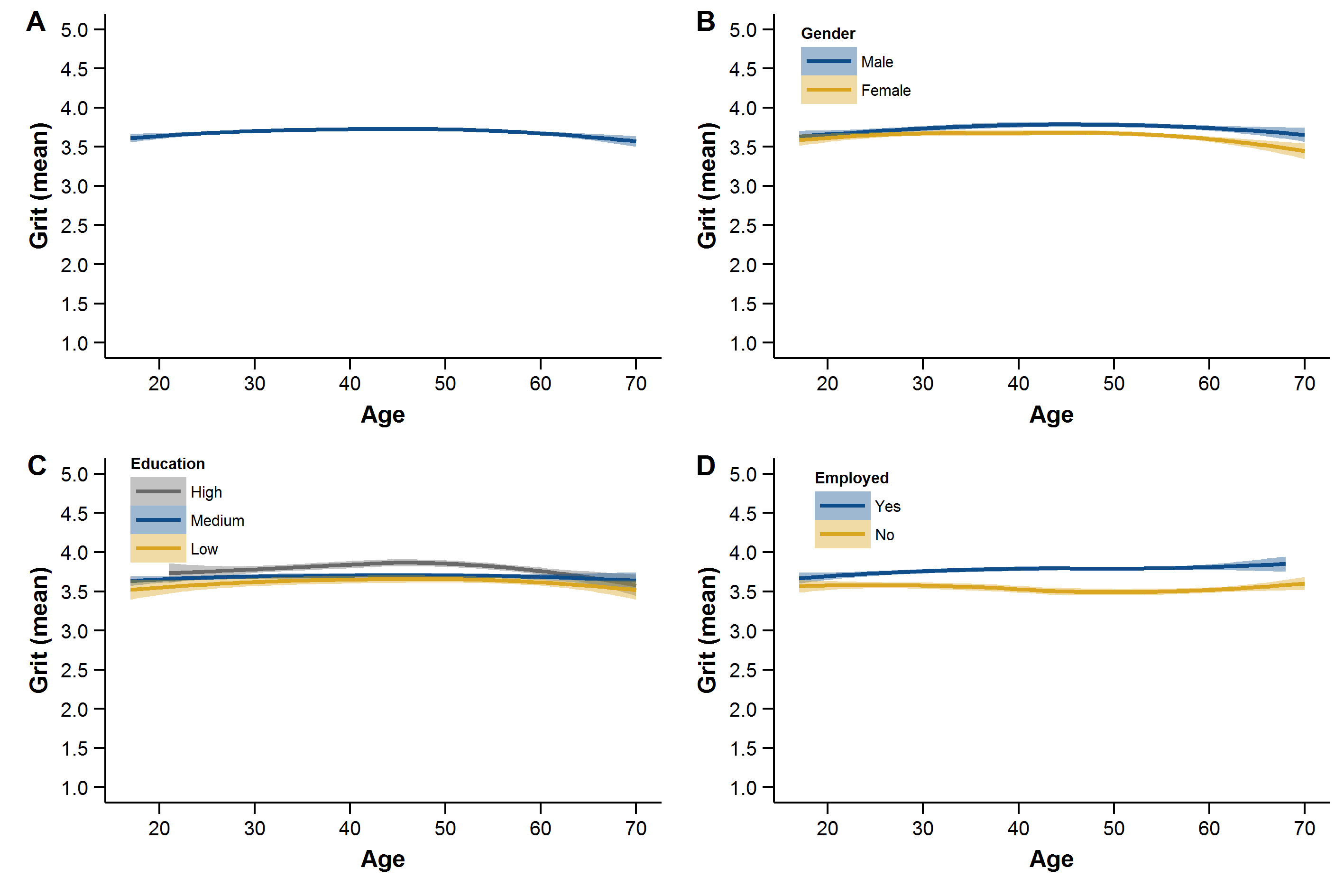


**S1 Fig. Age profiles of grit.** LOESS curves (A) for the full sample, (B) by gender, (C) educational attainment, and (D) employment status. Values on the *Y* axis are manifest scale means in the original item metric ranging from 1 (*not at all*) to 5 (*to a very large extent*). The manifest scale means correlated at *r* = .94 with the factor score estimates from the MIMIC model.
