# Supplementary material for: Grit (effortful persistence) can be measured with a short scale, shows little variation across socio-demographic subgroups, and is associated with career success and career engagement
Source: PLoS One. 2019 Nov 27;14(11):e0224814. doi: 10.1371/journal.pone.0224814 (PMC6881019; doi:10.1371/journal.pone.0224814)
Supplement: S1 Appendix — (DOCX) [file pone.0224814.s006.docx]

# S1 Appendix. Correlations of the Short Conscientiousness Instrument Used in Study 2 with a Full-Length Conscientiousness Scale and its Facets.

The correlations reported here are based on data from a German validation study of the BFI-2, carried out in December 2016 by a commercial online access panel [1]. The quota sample (based on age, gender and education) comprised *N* = 1,224 adults aged 18–65 years (*M* = 43.40 years), of which 50% were female. Respondents filled in the BFI-2 [2], which measures three conscientiousness facets with a total of 12 items. In addition, a subsample of respondents (*n* = 353) also filled in the BFI-S [3], which measures conscientiousness with three items (this is the measure used in the PIAAC-L survey). Using the sum scores per domain and facet, we computed the manifest correlations of BFI-S conscientiousness with BFI-2 conscientiousness as well as its facets. By means of confirmatory factor analyses (one with BFI-S conscientiousness and BFI-2 conscientiousness as latent variables; another with BFI-S conscientiousness and the three BFI-2 conscientiousness facets as latent variables), we estimated the latent-variable correlations.

**References**

[1] Danner, D., Rammstedt, R., Bluemke, M., Lechner, C. M., Berres, S., Knopf, T., Soto, C. J., & John, O. P. (2019). Das Big-Five Inventar 2: Validierung eines Persönlichkeitsinventars zur Erfassung von 5 Persönlichkeitsdomänen und 15 Facetten [The German Big-Five Inventory 2: Measuring 5 personality domains and 15 facets]. Advance online publication. *Diagnostica, 65*(3), 121–132. <https://doi.org/10.1026/0012-1924/a000218>

[2] Soto, C. J., & John, O. P. (2017). The next Big Five Inventory (BFI-2): Developing and assessing a hierarchical model with 15 facets to enhance bandwidth, fidelity, and predictive power. *Journal of Personality and Social Psychology*, *113*(1), 117–143. <https://doi.org/10.1037/pspp0000096>

[3] Schupp, J., & Gerlitz, J.-Y. (2014). Big Five Inventory-SOEP (BFI-S). In *Zusammenstellung sozialwissenschaftlicher Items und Skalen (ZIS)*. <https://doi.org/10.6102/zis54>
